# Early Surfactant Therapy With Nasal Continuous Positive Airway Pressure or Continued Mechanical Ventilation in Very Low Birth Weight Neonates With Respiratory Distress Syndrome

**DOI:** 10.5812/ircmj.12206

**Published:** 2014-04-05

**Authors:** Bita Najafian, Seyed Hasan Fakhraie, Seyed Abulfazl Afjeh, Mohammad Kazemian, Majid Shohrati, Amin Saburi

**Affiliations:** 1 Department of Pediatrics, Faculty of Medicine, Baqiyatallah University of Medical Sciences, Tehran, IR Iran; 2 Department of Pediatrics, Faculty of Medicine, Shahid Beheshti University of Medical Sciences, Tehran, IR Iran; 3 Chemical Injuries Research Center, Baqiyatallah University of Medical Sciences, Tehran, IR Iran

**Keywords:** Respiration, Artificial, Infant, Newborn, Respiratory Distress Syndrome, Newborn

## Abstract

**Background:**

Various strategies have been suggested for the treatment of respiratory distress syndrome (RDS).

**Objectives:**

The aim of this study was to compare the efficacies of two common methods of RDS management among neonates with low birth weight.

**Patients and Methods:**

A cohort study was conducted on 98 neonates with definite diagnosis of RDS during 2008-2009. The neonates were divided into two groups by a blinded supervisor using simple randomization (odd and even numbers). Forty-five cases in the first group were treated with intubation, surfactant therapy, extubation (INSURE method) followed by nasal continuous positive airway pressure (N.CPAP) and 53 cases in the second group underwent intubation, surfactant therapy followed by mechanical ventilation (MV).

**Results:**

Five (11.1%) cases in the first group and 23 (43%) cases in the second group expired during the study. The rates of MV dependency among cases with INSURE failure and cases in the MV group were 37% and 83%, respectively (P < 0.001). Birth weight (BW) (P = 0.017), presence of retinopathy of prematurity (P = 0.022), C/S delivery (P = 0.029) and presence of lung bleeding (P = 0.010) could significantly predict mortality in the second group, although only BW (P = 0.029) had a significant impact on the mortality rate in the first group. Moreover, BW was significantly related to the success rate in the first group (P = 0.001).

**Conclusions:**

Our findings demonstrated that INSURE plus NCPAP was more effective than the routine method (permanent intubation after surfactant prescription). In addition, the lower rates of mortality, MV dependency, duration of hospitalization, and complications were observed in cases treated with the INSURE method compared to the routine one.

## 1. Background

Mortality and morbidity rate in children with very low birth weight (VLBW) and prematurity remains high in spite of developments in therapeutics and instrumentation especially during the recent years (1). Respiratory distress syndrome (RDS) is one of the most common and fatal complications in premature neonates. Definite pathogenesis of this disease is the deficiency of surfactant due to prematurity of alveolar cells. Therefore, exogenous surfactant therapy is the main treatment (2, 3). A major issue in surfactant therapy among premature neonates is the time and method of prescription. Surfactant is usually prescribed via naso/oro tracheal tube, while it is necessary for neonates to be intubated for this method (3, 4). On the other hand, orotracheal intubation is one of the important risk factors for pneumonia, bronchopulmonary dysplasia (BPD) and it can increase the duration of hospitalization (5). In addition to pneumothorax, bronchopleural fistula, and development of nosocomial pneumonia, and ventilator dependency were reported in neonates intubated for long time (6).

Various protocols have been proposed to decrease the duration of intubation. One is the INSURE protocol which includes intubation, surfactant prescription, and extubation followed by nasal continuous positive airway pressure (NCPAP) (7). This protocol makes these neonates less exposed to the complications of intubation versus routine protocol including intubation, surfactant prescription, and mechanical ventilation (MV) (8). Moreover, there are a few reports that confirmed the efficacy of early treatment with surfactant and early use of NCPAP to reduce the need for intubation and continues MV and its complications (9). In addition, there are some evidence indicating the benefit of MV for neonate with irregular and non-spontaneous respiration, also the efficacy of CPAP in these patients has been reported (10). Previous studies showed the advantages of both protocols to reduce mortality and morbidity of children with RDS (11-13), but there are some queries on the efficacy of these protocols in children with RDS and VLBW. It seems that more clinical studies are required to compare the efficacy of the two mentioned protocols.

## 2. Objectives

The aim of this study was to compare the efficacy of these two methods (INSURE plus NCPAP versus intubation, surfactant therapy and maintained MV) for the treatment of VLBW neonates with RDS.

## 3. Patients and Methods

### 3.1. Study Population

A cohort study was conducted on neonates born between 2008 and 2009 in a tertiary care hospital (Mofid Hospital) in Tehran, Iran. Newborns with clinical symptoms of RDS and birth weights less than 1500 g (VLBW) were consecutively included. RDS was diagnosed by one neonatologist according to clinical symptoms (cyanosis, grunting, retractions, and tachypnea), radiographic studies (ground glass or air bronchograms pattern in CXR) and physical examination (14). Patients with congenital anomalies, pulmonary structure abnormalities, chromosomal abnormalities, any evidence of sepsis (maternal and neonatal confirmed by positive blood culture) and congenital heart diseases were excluded. Among 893 cases, finally, 98 neonates with RDS score more than 6, i.e. moderate to severe degree (Table 1), were included (15). The two groups were matched regarding the severity of RDS, maternal situations, nutritional status of the parturient, indications for LSCS, lack of evidence for meconium aspiration and other confounding variables as seen in Table 2 and presented P values.

**Table 1.** RDS Scoring (15)

Score	0	1	2
**Respiration rate, breath/min**	≤ 60	60-80	≥80
**FIO**_**2**_	> 40%	21-40%	< 21%
**Retraction**			
Intercostals	None	Moderate to severe	mild
Sub costal	None	Moderate to severe	mild
**Grunting**	None	Audible with stethoscope	Audible without stethoscope

**Table 2.** Baseline Characteristics ^a,b,c,d^

Variable	First Group	Second Group	P Value
**Gender**			0.934
Male	25 (46.3)	29 (53.7)	
Female	20 (45.5)	24 (54.5)	
**Delivery**			0.054
NVD	6 (24)	19 (76)	
C/S	39 (53.4)	34 (46.6)	
**Parity**			0.211
Primiparity	31 (50.8)	30 (49.2)	
Multiparity	14 (37.8)	23 (62.2)	
**Birth Weight, g**	1171 ± 218	1157 ± 210	0.744
**Gestational Age, wk**	29.60 ± 2.28	28.94 ± 2.08	0.140

^a^ Abbreviations: C/S, cesarean section; NVD, normal vaginal delivery.

^b^ First group underwent INSURE and then NCPAP.

^c^ Second group underwent intubation, surfactant therapy and then MV.

^d^ Data are presented as No. (%).

### 3.2. Intervention

**Figure 1.**
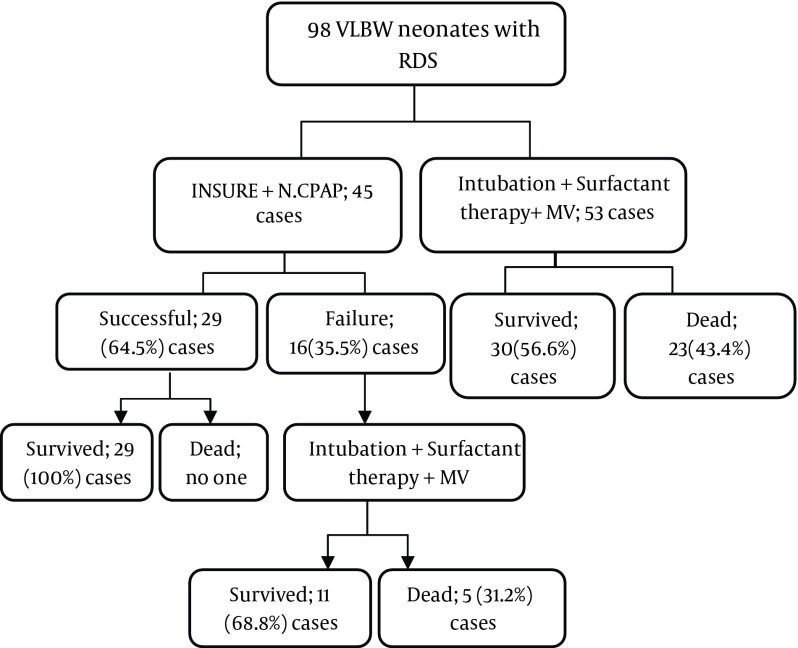
Flow Chart of the Study Design RDS; Respiratory distress syndrome, MV; mechanical ventilation, INSURE; intubation plus surfactant therapy and extubation, VLBW; very low birth weight, N.CPAP; Nasal Continuous Positive Airway Pressure.

To prevent the selection bias, the patients were divided into two groups by one supervisor using simple randomization method (odd and even numbers); newborns in the first group underwent INSURE and then NCPAP (with 4 cm/H_2_O), and those in the second group underwent intubation, surfactant therapy and then MV. One-hundred mg/kg surfactant (Intratracheal Suspension of Survanta, Beractant, Columbus, Ohio, USA) warmed to 37 ^o^C was prescribed at each injection. At every time after the treatment, patients in the first group that their RDS symptoms were resolved or had a suitable arterial blood gas (ABG) analysis and also acceptable control chest X-Ray were considered as successful INSURE. If their symptoms remained stable with FiO_2_ < 40, positive end expiratory pressure (PEEP) < 5 cm/H_2_O and in ABG analysis; PaCO_2_ < 60 mmHg, PaO_2_ > 50 mmHg and PH > 7.25, they were considered stable and their treatment were continued by oxygen-hood and followed up. In contrast, patients in the first group who had O_2_ saturation under 85%, and in ABG analysis as PaCO_2_ > 60 mmHg, PaO_2_ < 50 mmHg and PH < 7.2 while the PEEP increased to 6 cm H_2_O underwent intubation and MV and they entitled as INSURE failure group (16). Additional dose of surfactant was prescribed when FiO_2_ was more than 40 and required O_2_ saturation to increase to 85%. The study protocol was shown in Figure 1.

### 3.3. Statistical Analysis

Quantitative data were extracted as mean ± standard deviation (SD). Statistical analyses were performed by using SPSS software (16th edition, SPSS Inc., Chicago). Normal distribution of data was checked by using Kolmogorov-Smirnov test, and those variables without normal distribution were tested by nonparametric method. Independent t-test, Mann-Whitney U test and ANOVA (for two or more independent groups) were used for analyses. Variables with P-value less than 0.2 in univariate analysis were entered the logistic regression model. P-value < 0.05 was considered statistically significant. In addition, statistician was blind to the groups. Dependent variables such as duration of hospitalization, mortality rate, complications of treatment including pneumothorax and ventilator dependency were considered as the efficacy of treatment. The intervention began after obtaining informed consent signed by the parents. There was no extra charge imposed to the patients’ parents. The study protocol and proposal were approved by the Ethics Committee of Medical School of Shahid Beheshti University of Medical Sciences at 2009 (approval code: No.221). Parents of neonates could leave the study at any time. Data was inserted in checklist using blind codes by keeping the privacy of patients.

## 4. Results

Ninety-eight neonates (45 in the first group; INSURE+NCPAP and 53 in the second group (intubation and MV)) completed the study. The overall mean ± SD of gestational age was 29.24 ± 2.19 weeks and the mean birth weight was 1.164 ± 2.13 g. In general, 54 (55.1%) cases were male, 73 (74.4%) neonates were born via elective cesarean section (C/S) and 61 (62.2%) mothers were primiparous. Twenty-five cases (55.5%) in the first group were male compared to 29 (54.7%) in the second group. In addition, six cases (13.3%) in the first group were born via NVD compared to 19 (35.8%) in the second group. The two groups were matched regarding demographic and clinical characteristics (Table 2). A single dose of antenatal corticosteroids was administered for all cases. The most common gestational complication was gestational hypertension (19.1%) followed by prolonged rupture of membrane (4%) and placenta abruption (2%). The mean Apgar score between the two groups was not statistically different (P = 0.172).

There was no significant difference in gestational age (P = 0.140), birth weight (P = 0.740), gender (P = 0.934) and time of surfactant prescription (P = 0.521) between the two groups (Table 2). The success rate in INSURE group was 64.4% (29 cases), while condition of 16 (35.6%) cases became worse and they underwent MV after INSURE failure. In MV group, 30 cases (56%) survived at the end of study, while 5 (11.1%) cases in the first group and 23 (43%) cases of the second group died during the study. The most common cause of death was respiratory failure (46.42%), followed by sepsis (42.85%) and NEC (10.71%). INSURE method in all expired newborns of the first group was failed and they underwent respiratory support by MV (Figure 1).

A significant difference was found between the two subgroups (successful and failure) among the patients of the first group (INSURE method) regarding birth weight (P < 0.001, r = -5.20), gestational age (GA) (P: 0.001, r= -3.655), N.CPAP duration (P < 0.001, F = 18.531), lung bleeding (P < 0.001, odds ratio (OR): 0.237, confidence interval (CI) [95%]: 0.134-0.419), presence of necrotizing enterocolitis (NEC) (P = 0.012, OR:0.293, CI [95%]: 0.182-0.471) and mortality (P = 0.004, OR:0.275, CI[95%]: 0.166-0.455) (Table 3). GA was the only variable associated with duration of hospitalization between the two subgroups, successful and unsuccessful INSURE (P = 0.001). Moreover, GA (P < 0.001, r = 3.669), birth weight (P < 0.001, r= 4.358), delivery via C/S (P = 0.048, OR: 1.889, CI [95%]: 1.029-3.468), lung bleeding (P < 0.001, OR: 6.923, CI [95%]: 2.547-18.817) and presence of NEC (P = 0.015, OR:4.333, CI[95%]: 1.243-15.104) were significantly different between neonates expired or survived (Table 4). In the INSURE group, prevalence of pneumothorax (P = 0.033, OR: 0.178, CI [95%]: 0.037-0.750), lung bleeding (P = 0.037, OR: 0.358, CI [95%]: 0.134-0.961), mortality (P < 0.001, OR: 0.163, CI [95%]: 0.056-0.479) and duration of need to O_2_ support (P = 0.018, r= -2.399, CI [95%]: -13.24-1.25) were significantly lower than the MV group, and there were no significant differences for other items between the two groups (P > 0.05).

Variables with P < 0.2 in univariate analysis were included for multivariate analysis. Logistic regression test indicated that only birth weight (P = 0.001, EXP [β]: 0.009, CI [95%]: 1.003-0.014) had a significant association with success rate in the first group. In addition, birth weight (P = 0.017, EXP [β]: 0.995, CI [95%]: 0.991-0.999), presence of retinopathy of prematurity (ROP) (P = 0.022, EXP [β]: 0.011, CI [95%]: 0.000-0.516), delivery via C/S (P = 0.029, EXP [β]: 0.129, CI [95%]: 0.021-0.810) and presence of lung bleeding (P = 0.010, EXP [β]: 14.220, CI [95%]: 1.862-18.603) could significantly predict death in the second group, although only birth weight (P = 0.029, EXP [β]: 0.993, CI [95%]: 0.987-0.999) had a significant influence on the mortality rate in the first group. The incidence of MV dependency (that was defined at least more than 2 days) in INSURE failure subgroup compared to the MV group was 37% and 83%, respectively, which indicated a statistically significant difference (P < 0.001).

**Table 3.** Comparison of Patients Characteristics Between the Two Subgroups of INSURE Group ^a, b^

Variable	Successful	Failure	P Value	Duration of Hospitalization	P Value
**Delivery**					0.960
NVD	4 (66.7)	2 (33.3)	0.995	31.8	
C/S	25 (64.1)	14 (35.9)		38.3	
**Gravidity**					0.227
Primiparity	22 (71)	9 (29)	0.639	35	
Multiparity	7 (50)	7 (50)		42	
**Gender**					0.166
Male	17 (68)	8 (32)	0.362	33.8	
Female	12 (60)	8 (40)		42.1	
**Twins**					0.897
Singleton	12 (52.2)	11 (47.8)	> 0.999	33.8	
Twin	17 (77.3)	5 (22.7)		37	
**G-HTN**					0.829
Yes	4 (57.1)	3 (42.9)	0.423	37	
No	25(65.5)	13 (34.2)		39	
**Birth weight, g**					0.164
< 1000	1 (11.1)	8 (88.9)	0.002 ^c^	46.6	
1000-1250	12 (70.6)	5 (29.4)		39	
1250-1500	16 (84.2)	3 (15.8)		31.7	
**Gestational age, wk**					0.001
< 28	5 (33.3)	10 (66.7)	0.465	51.5	
28-32	20 (76.9)	6 (23.1)		31.9	
33-37	4 (100)	0 (0)		20.7	
**Surfactant Dose**					0.756
Single	28 (68.3)	13 (31.7)	> 0.999	37	
Multiple	1 (25)	3 (75)		34	
**Time of surfactant administration, hr**					0.256
< 2	8 (72.7)	3 (27.3)	0.586	29	
2-4	12 (66.7)	6 (33.3)		41	
4-6	14 (44.4)	5 (55.6)		46	
7-12	1 (49.4)	1 (50.6)		25	
13-24	4 (80)	1 (20)		30	
**Duration of N.CPAP, hr**					0.195
< 6	8 (100)	0 (0)	0.586	26	
7-12	3 (75)	1 (25)		38	
13-24	8 (88.9)	1 (11.1)		35	
24-48	7 (63.6)	4 (36.4)		34	
> 48	4 (23.1)	1 (76.9)		47	
**Pneumothorax**					0.919
Yes	0 (0)	2 (100)	0.212	37	
no	29 (67.4)	14 (32.6)		37	
**Sepsis**					0.641
Yes	6 (60)	4 (40)	> 0.999	37	
No	23 (65.7)	12 (34.3)		33	
**NEC**					0.734
Yes	0 (0)	4 (100)	0.555	37	
No	29 (70.7)	12 (29.3)		33	
**ROP**					0.734
Yes	2 (66.7)	14 (33.3)	0.304	37	
No	8 (33.3)	2 (66.7)		33	
**Lung bleeding**					0.735
Yes	6 (60)	4 (40)	0.210	37	
No	23 (65.7)	12 (34.3)		39	

^a^ Abbreviations: C/S, cesarean section; G-HTN, gestational hypertension; N.CPAP, nasal continuous positive airway pressure; NEC, necrotizing enterocolitis; NVD, normal vaginal delivery; ROP, retinopathy of prematurity.

^b^ Data are presented as No. (%).

^c^ Statistically significant.

**Table 4.** Comparison of Patients Characteristics Between the Two Sub-Groups of MV Group ^a,b^

Variable	Survived	Dead	P Value	Duration of Hospitalization	P Value
**Delivery**			0.111		0.554
NVD	8 (42.1)	11 (57.9)		40.3	
C/S	22 (64.7)	12 (35.3)		35.9	
**Gravidity**			0.960		0.147
Primiparity	14 (46.7)	16 (53.3)		33	
Multiparity	16 (69.6)	7 (30.4)		43.3	
**Gender**			0.745		0.381
Male	17 (58.6)	12 (41.4)		34.6	
Female	13 (54.2)	11 (45.8)		40.9	
**Twins**			0.413		0.456
Singleton	19 (52.8)	17 (47.2)		35.6	
Twin	11 (46.7)	6 (35.3)		41.3	
**Birth weight, gr**			0.002 ^c^		0.576
< 1000	4 (28.6)	10 (71.4)		33.5	
1000-1250	10 (47.6)	11 (52.4)		42	
1250-1500	16 (88.9)	2 (11.1)		35.2	
**Gestational age, wk**			< 0.001		0.335
< 28	7 (29.2)	17 (70.8)		39.6	
28-32	22 (84.6)	4 (15.4)		37.9	
33-37	1 (33.3)	2 (66.7)		16.3	
**Surfactant Dose**			0.177		0.186
Single	25 (61)	16 (39)		40	
Multiple	5 (36)	7 (64)		28	
**Time of surfactant administration, hr**			0.850		0.724
< 2	14 (58.3)	10 (41.7)		41	
2-4	6 (60)	4 (40)		37	
4-6	4 (66.7)	2 (33.3)		41	
7-12	5 (45.5)	6 (54.5)		29	
13-24	1 (100)	0 (0)		37	
**Pneumothorax**					0.303
Yes	6 (54.5)	5 (45.5)	> 0.999	35	
no	24 (57.1)	18 (42.9)		44	
**Sepsis**			0.194		0.722
Yes	13 (68.4)	6 (31.6)		38	
No	17 (50)	17 (50)		35	
**NEC**			0.272		0.616
Yes	3 (37.5)	5 (62.5)		38	
No	27 (60)	18 (40)		33	
**ROP**			0.100		0.001
Yes	2 (66.7)	1 (33.3)		82	
No	28 (56)	22 (44)		34	
**Lung bleeding**			0.014		0.475
Yes	6 (43.3)	12 (66.7)		39	
No	24 (68.6)	11 (31.4)		33	

^a^ Abbreviations: C/S, cesarean section; G-HTN, gestational hypertension; N.CPAP, nasal continuous positive airway pressure; NEC, necrotizing enterocolitis; NVD, normal vaginal delivery; ROP, retinopathy of prematurity.

^b^ Data are presented as No. (%).

^c^ Statistically significant.

## 5. Discussion

Our findings showed that INSURE method with N.CPAP was safer and more effective than the routine method (Intubation plus MV after surfactant therapy) in VLBW children with RDS. Additionally, INSURE method was associated with lower mortality, morbidity rate and less dependency to MV during hospitalization, and the mentioned factors could be considered as success (efficacy) criteria compared with the previous study regarding efficacy and MV dependency.

Previous reports compared the efficacy and safety of various methods of RDS management. There is a controversy to choose the best method for pulmonary support of neonates with VLBW and RDS. However, the efficacy of N.CPAP compared to permanent MV has been reported in previous studies but it is not clear when and how it should be used (13, 17-21).

Nowadays, positive airway pressure including N.CPAP has been used for the treatment of neonates with RDS after surfactant therapy. Ancora et al. investigated the efficacy of bi-level positive airway pressure (Bi-PAP) for neonates after INSURE failure. They found that MV dependency was significantly lower in the BiPAP group compared to control group consistent with our results (22). In addition, Surfactant therapy was reported with or without NCPAP.

In a study by Rojas et al., premature neonates with RDS were divided into two groups who received surfactant with or without NCPAP. They finally reported that MV dependency decreased from 39% in neonates treated with NCPAP to 26% in neonates treated by early surfactant, which is deferent with our study protocol (23). Gopel et al. prescribed surfactant in addition to CPAP in preterm neonates with spontaneous breathing and RDS. They found that these patients had a decreased rate of MV dependency in comparison with control group, which is similar to our findings (24).

In the present study, repeated administration of surfactant versus single dose was not effective enough to change the rate of INSURE failure. In contrary, Dani et al. demonstrated that multiple INSURE strategy could increase the success rate of INSURE and might prevent MV dependency, but the method of this study was different with our one in case selection which can justify these differences (7, 25). Rate of INSURE failure in the present study was 35.6% similar to other studies (26). Arterial pressure of carbon dioxide, mean arterial-to-alveolar oxygen pressure ratio and severe radiological abnormalities were previously reported as risk factors for INSURE failure which are not coherent with the results of our study (26). In this study and after adjusting confounding variables, birth weight was found as the only risk factor for INSURE failure which is consistent with Dani et al.’s study results (25). In addition, Cherif et al. demonstrated that the mortality rate was significantly higher among infants with INSURE failure and our study findings confirmed this result (26).

MV dependency (or Need to long time MV) is one of the dangerous complications of MV among infants with RDS. Transient intubation and CPAP can somewhat reduce this problem. Dani et al. showed that neonates treated with transient intubation and NCPAP rather than neonates treated with continuous MV had higher rate of MV dependency and this finding is concordant with our result (19). Additionally, in the present study, the rate of oxygen therapy dependency in INSURE group was less than the rate in MV group and this finding is similar to previous results (20).

On the other hand, previous reports evaluated the time of surfactant prescription (early or late) and some of them showed that the early surfactant therapy is not always effective enough to prevent MV dependency (27). Escobedo et al. showed that the duration of hospitalization and adverse outcomes of preterm neonates with RDS treated with early intubation and surfactant administration were not significantly different with patients treated with routine protocol (20). In a systematic review, Stevens et al. concluded that the frequency of MV dependency, BPD and air leak syndrome in a group of neonates treated with early surfactant therapy and NCPAP were less than a group treated with delayed surfactant therapy and MV, and this finding is similar to our findings (8).

We also found that birth weight, rate of ROP, delivery via C/S and presence of lung bleeding could be associated with neonatal mortality seen in the MV group and is concordant with previous reports (28, 29). CPAP can enhance the frequency of hospitalization complications such as NEC which is not in agreement with our results (30).

There were some limitations in this study; first, our sampling method was consecutive method and our allocation method was simple allocation (using even and odd numbers). We had to use the most available methods for randomization and allocation. Second, we did not follow these cases for long time. Third, the follow-up duration should be more similar in both groups. There were some confounding factors such as age and Apgar score, which were neutralized by logistic regression models.

The present study conducted to reply this important question that; “whether early administration of surfactant followed by quick extubation and NCPAP is better than surfactant therapy followed by continues MV”. Our findings on preterm VLBW infants with RDS declared that INSURE method is related to lower need to supplementary O_2_, mortality and the rate of pneumothorax compared to the routine strategy (surfactant therapy followed by MV). In addition, we concluded that BW is a valuable predictor for efficacy of treatment regardless of treatment methods in VLBW neonates with RDS.
